# Selections and Abstracts

**Published:** 1906-02

**Authors:** 


### SELECTIONS AND ABSTRACTS.

### RESULTS OF TIMID EMPLOYMENT OF DIPHTHERIA ANTITOXIN.*

It is not too sweeping a statement to say that antitoxin will cure every case of diphtheria when administered early and in proper doses. The large volume of literature upon the subject abundantly justifies this assertion. The proof of the fact is found in both laboratory and clinical evidence of the most unique character. Either is complete in itself and sufficiently conclusive to bring conviction to any unbiased mind. Considered together they constitute a chain of evidence stronger than that supporting any other remedial agent ever employed.

Were it necessary to offer an apology for the already enormous volume of antitoxin literature, the fact that the present greatly reduced mortality from diphtheria is still too high, owing to late and inadequate use of antitoxin, would be sufficient. To demonstrate this fact and to present a plea for the employment of antitoxin at the earliest possible moment is the purpose of this paper. But there are other and minor reasons for keeping the subject fresh in the stream of current medical literature. The avowed enemy of antitoxin is neither dead nor asleep. While the voices of the most prominent opponents five years ago are now silent, there are still cries to be heard which should be met with at least a plain statement of facts.

The source and nature of antitoxin are so unique and absolutely without precedent in medical science that great opposition to its introduction was to be expected. Greater progre-s, however, was made in five years with antitoxin than in half a century with Jenner's vaccine virus. The difference is due to increased intelligence and enlightenment of the medical profession. The question of dosage at the bedside had to be worked out experimentally.

---

*Read by request before the Lehigh County Medical Society, Allentown, Tuesday, November 14, 1905, by I. Newton Snively, A.M., M.D., Dean and Professor of Materia Medica, Therapeutics and Clinical Medicine in the Medical Department of the Temple College, Philadelphia.

In the laboratory, on the other hand, it is a matter of mathematics and great accuracy was possible from the first. Every factor involved could be ascertained with scientific accuracy. A given number of toxic units requires for its neutralization a definite number of antitoxin units in laboratory work.

In the clinical use of antitoxin such action will never be possible. We have not the means at hand for even approximately estimating the toxins present in any given case of diphtheria, hence the necessity for more or less experimentation.

In the determination of the proper dose there were several powerful factors that made for small quantities administered at fairly long intervals. Among these were timidity, the source of the antitoxic fluid and the initial cost of the remedy. The vile epithets applied to it and the spirited opposition of a few leading clinicians at home and abroad no doubt did much to increase the timidity of many physicians. Then again our early conception of diphtheria required careful revision. Questions of case malignancy could be properly solved only as other problems were rendered simple. The latter had to do chiefly with different degrees of toxicity of the products of different infections of the diphtheria bacillus and the role of conjoint, added or secondary infections. The malignancy of a particular case was found to depend less upon the extent and character of the local lesion than was once supposed.

Mild cases, that is cases of very recent development, and all cases in which only a small quantity of a moderately virulent toxin had been absorbed, yield promptly and charmingly to so small a dose as five hundred units repeated in eight or ten hours when indicated. These hitherto unparalleled therapeutic effects are the most interesting facts in the medical literature of many centuries. They produced results in the treatment of diphtheria of directly opposite natures. While reducing the general mortality of this once most dreaded of all diseases to less than half its former rate, these results inspired confidence in doses entirely too small in cases which are not mild. In consequence of the latter many cases which would no doubt have yielded to much larger quantities of antitoxin with equal facility with the manifestly mild cases, have gone to swell the rate of mortality.

During the past four years we have learned two important lessons in treatment of diphtheria; namely, *the absolute harmlessness of antitoxic serum and the superiority of large initial doses administered at the earliest possible moment.* The latter when generally utilized, will further reduce the diphtheria mortality to much less than five per cent. That the death-rate from this disease is still too high, in view of the specific powers of antitoxin, is susceptible of proof. This, indeed, must be evident to every fair mind that takes pains to inquire into recent leading reports of large numbers of cases treated in private and hospital practices.

If doses containing enough antitoxic units to neutralize the entire quantity of toxins in the system could be administered in each and every case of diphtheria before serious harm has been done by the disease, the mortality would be nil. That would seem self-evident. It is, however, daily demonstrated in the antitoxin laboratory. It is also abundantly proved in actual practice in the management of all well-treated cases. The change in the clinical picture following the administration of an adequate dose of antitoxin, when the symptoms produced by tissue destruction and added infection do not overshadow all others, is a complete reversal, explainable only by supposing a complete removal of the cause.

The fact that early treatment requires smaller doses to accomplish the same results as larger doses given at later stages in the disease is equally well established. In all statistics tabulated with respect to the period when treatment was inaugurated, the rate of mortality invariably increases rapidly from first day cases upward. Another fact that is not not clearly established in medical literature, and yet equally demonstrable, is that one large dose of antitoxin will prove successful in more instances than the same number of units given in two or three doses at intervals of six to ten hours. By the latter method much valuable time is lost to the patient. Time is permitted for unneutralized toxins to do their full work and other infections to be added.

Barring the lack of opportunity to form an early correct diagnosis, the most prolific cause of unwarranted results from antitoxin treatment is timidity on the part of the attending physician. Next to this must be placed a lack of an adequate conception of the nature and value of the remedy. Prompted by fear of the undesirable results, excessive cost or what not, physicians are still too prone to wait developments with a view of using antitoxin only in *severe cases.* They are like the fire company that would wait to see whether it was an ordinary fire or a conflagration that is to be extinguished before applying water. The utter folly ot such delay is too obvious to require further detailing. There is nothing in the remedy to preclude its administration in enormous quantities while there is every indication needful in any case of diphtheria for the earliest possible administration of an adeqate dose of antitoxin. Such a dose, it may be stated, is enough to neutralize all the toxins in the system at the time. It may contain ten to fifteen thousand units. I have given as much as twenty-two thousand, five hundred units in one case in less than forty-eight hours with complete recovery. Dr. McCallom, Boston, reports a total administration of one hundred thousand units in a single case. While such enormous quantities should never be required, the fact that they have been administered is a practical demonstration of the blandness of antitoxin serum; and the fact that they have been found necessary to a cure should constitute a powerful incentive to early treatment. Again the authentic record of the administration of such doses without untoward effects and, what is more, with the result that life was saved, should embolden the most timid physician.

What if urticaria or arthralgia or abscess results so long as life can be saved? The elements of idiosyncrasy, we can not control, nor should this fact control the physician in the use of our best remedy against diphtheria. Certain it is that no fact in medicine is more clearly demonstrated than that initial doses of twenty to thirty thousand units of antitoxin can be administered with absolute impunity. Moreover, the results of the use of such large doses, ten thousand units and over, justify their more frequent employment as early as possible in combating manifestly desperate cases.

The physician who is fully awake to the value of antitoxin finds no difficulty in obtaining parents' consent to treat a case properly.

My total number of cases of diphtheria to date treated with antitoxin is one hundred and forty-seven. All were treated in private practice in the families of the well-to-do and wealthy. The initial case in this series was the first case of diphtheria to be treated with antitoxin in Philadelphia and in the State of Pennsylvania. As a rule the cases were seen early and treatment begun promptly. Several cases seen in consultation practice required especially large doses. There were but two deaths in the entire series of one hundred and forty-seven cases. These belong to the early years of antitoxin treatment. One of the cases was laryngeal in type and of marked severity. The parents refused to have the remedy used till the case was hopeless. When permitted to have recourse to anything whatever the case was far beyond the reach of what was then regarded as heroic doses. The other fatal case was tonsillar in type and of frightful malignancy. It was of the class of cases in which in subsequent years I learned to resort to the enormous doses above stated.

I have not had a death from diphtheria during the last seven years. All cases are treated with antitoxin in full doses, repeated every six or eight hours, according to the effect produced. My initial doses range from two thousand units upwards and treatment is inaugurated as early as possible. The size of the dose to be employed at the beginning of treatment or subsequently, I determine especially for each particular case and aim always to give more than may be required rather than the exact quantity. The old adage, "Aim above the mark you wish to hit," is applicable in antitoxin treatment as nowhere else in medicine.

The need for a second dose, and whether this dose should be larger or smaller than the first, must be determined by a careful study of the aspects of the case. An adequate dose never fails to show adequate results within six or eight hours. The absence of such results is always an indication for the use of larger quantities. The condition of the patient after six hours may show that the case was more severe than was at first believed, in which instance I resort to a larger dose of antitoxin. A recent case which required these larger quantities was that in which I employed a total of twenty-two thousand five hundred units. The case was seen in consultation after it had progressed several days. It was laryngeal in type and had some of the features of a severe case of broncho-pneumonia, for which the patient was at first treated. The case was extremely malignant and hopeless to every earlier method of treatment, as well as to ordinary antitoxin treatment. All the extraordinary respiratory muscles were in full play and the child had repeated unconsciousness from exhaustion. Only the most heroic dosage held out any hope. My largest dose proved inadequate. The quantity was largely increased in subsequent doses so that within nearly forty-eight hours the above total administered. The results justified the treatment.

Another case similar in type but of shorter duration was likewise treated in consultation. The child was two and one-half years old. He was unconscious when first seen. Following the plan above indicated heroic doses were repeated till a total of fourteen thousand units were reached on the second day of treatment. The result showed the wisdom of the treatment in a complete recovery.

It is vain to calculate how many thousand lives could have been saved during the past ten years by the proper use of antitoxin to prevent and cure diphtheria. That would be figuring on the value of spilled milk. But we have a lesson of great and future value, and while there is a single case of diphtheria in the land it is the bounden duty of every physician who is convinced of the specific power of antitoxin to talk and teach till the entire profession is properly aroused.—*Pennsylvania Medical Journal.*

---

### SYMPTOMATOLOGY OF NEPHRITIS.

BY GEORGE L. COLE, M.D., LOS ANGELES, PROFESSOR OF MEDICINE IN THE COLLEGE OF MEDICINE, UNIVERSITY OF SOUTHERN CALIFORNIA.

I regret exceedingly that Dr. Lemke can not be here to speak of the symptomatology of nephritis, because I know full well his ability to present it in a clear and lucid manner. However, I am just as sure that Dr. Woods Hutchison, who is to follow me with a talk upon diagnosis, will be kind enough to correct any errors I may inadvertently make, and to supply any omissions of which I may be guilty.

The symptomatology of nephritis is so complete that it becomes quite necessary to make some classification of the different forms. A simple division which I am in the habit of making to my class, and which I think works out well practically, is that of, first, acute and second, chronic nephritis. The chronic forms being subdivided into (a) parenchymatous and (b) interstitial nephritis. I think the point made by some authors in speaking of subchronic rather than subacute nephritis, remembering that the division between acute and chronic has no definite line of demarkation, is well grounded.

I am quite aware that Councilman describes an acute interstitial nephritis, occurring usually in children after fever, and I am not unaware of the fact that several other forms of nephritis have been described, but I assume this simple division as a working basis for the symptomatology, for an approximate prognosis, and to help us gain a better understanding of the disease in its complexity.

I can not refrain from saying a word about the urinary findings, which are more or less constant in acute nephritis, showing usually a diminution in the amount, a high color, cloudiness, usually with a precipitate, a high specific gravity, much albumen, and all forms of casts and blood elements usually in abundance.

In teaching of the chronic forms, I try to impress upon my class the different urinary findings in the parenchymatous and interstitial forms. In the former, small quantities of urine of a high specific gravity, with albumen, more or less abundant, and in the latter form—interstitial nephritis—large quantities of urine of low specific gravity with various forms of casts, often not abundant, and with or without albumen present, but if present, usually in small quantities.

I am again not unmindful of the recent autopsy findings to which Cabot has drawn our attention in which the postmortem work was preceded by careful urinary examination extending over weeks, months, and in some cases years. His findings would tend to reject somewhat our preconceived notions as to what we should expect to find in the kidney as a sequel to certain urinary findings. While we all have confidence in the ability and integrity of Cabot, I can not but remember how easy it is to be led onward by enthusiasm when blazing a new path in medicine, and, for one, I shall await patiently further investigations in this direction before being willing to accept completely the upsetting of the result of work which has been going on for scores of years by careful men in clinical work who have been able also to observe their cases at autopsy.

Now, in acute nephritis, aside from the urinary findings, while we look upon edema and rapid anemia as perhaps the two most constant symptoms, we must not forget Osler's remark, that "the most intense nephritis may exist without anasarca." Furthermore we must not forget that in children the symptoms of acute nephritis may point rather to digestive derangements or brain disease than to nephritis, and thus remember the importance of careful urinary analysis in children.

It is perhaps in the chronic interstitial form that we find the complex symptomatology most marked, and it is quite impossible to get a clear understanding of the symptomatology of chronic interstitial nephritis without taking it up under the various systems of the body, such as the urinary, circulatory, respiratory, digestive and nervous systems, forgetting the importance of the eye and ear under the head of special senses. I shall say nothing more of the urinary system in this connection, as that, I think, should more properly come under diagnosis.

But there is much to be said with regard to the circulatory system. The increased arterial tension, with the resulting arterial changes and the changes brought about in the heart itself, really form the important part of this form of nephritis. But I think it is well for us to consider it in the light in which it is viewed by Strumpell. We shall then better understand its importance. He considers the increased arterial pressure, with its result upon the heart and blood-vessels, as a compensatory change, in the same manner as a hypertrophied left ventricle is a compensatory change in mitral regurgitation.

As some of the glomeruli become diseased, an increased arterial tension is necessary to procure elimination of the urinary elements through the remaining normal glomeruli. When this compensatory change becomes broken then the organism goes to pieces, as it does when the compensating heart becomes "broken."

Furthermore it is interesting to notice how Strumpell explains the fact that albumin is found in the urine sometimes, and not at others: namely, that when certain diseased glomeruli cease to functionate entirely, then the normal ones carry on the process and albumin is not eliminated. If we follow this a little further it will explain to us perhaps why a morning urine is frequently free from albumin, as that which is discharged in the morning is only what has remained in the bladder during the latter part of the night; remembering the frequency with which micturition occurs at night in this class of cases. The influence of the exertion of the previous day and of the evening meal has passed away. Therefore the albumin is not so frequently found in the morning's urine when we have a low arterial tension as it is in the afternoon when the arterial pressure is higher.

Considering the digestive system, I shall never forget the question which the late Alfred Loomis used to ask so frequently the members of his class as to what the first symptoms of chronic interstitial nephritis were likely to be, and woe unto the student who forgot to say "digestive disturbances." The dyspepsia, the uncontrollable vomiting, the coated, foul tongue, with breath of urinous odor, the sometimes profuse diarrhea must not be forgotten as possibilities along the line of observation.

As to the nervous system, headache, Cheyne-Stokes respiration in patients that are sometimes going about, and the possible cerebral apoplexy resulting from increased arterial tension and arterial changes are evidences which are only too frequent.

The respiratory system showing oppressed breathing, sometimes spoken of as uremic asthma, attacks of edema of the lungs, pleuritic effusions, complicating pneumonias, etc., bring a train of symptoms that must not be overlooked.

With regard to special senses, the buzzing and ringing of the ear with its annoyance, and the eye showing its changes by means of the ophthalmoscope, often lead the oculist and aurist to make the diagnosis by these symptoms alone.

Therefore let us remember that in order to work intelligibly along the lines of the symptomatology of nephritis we must not look upon nephritis as a disease having a first, second and third stage, as Bright and his followers did. But that we must take some working form of classification and study the symptoms along the lines of such a classification, considering them practically as different diseases.—*Southern California Practitioner.*

---

### THE MEDICAL DEPARTMENT OF THE ARMY.*

The readers of the *Medical Sentinel* scarcely need figures to convince them that the Medical Department of our Army is entitled to all the support it can receive in its efforts to raise the standard of its service. Yet, so important is the question, that we may be pardoned if we quote figures based upon the official records. There has rarely been a conflict of any duration in which at least four men have not perished from disease for every one from bullets. In the Russo-Turkish war, 80,000 men died from disease and 20,000 from wounds. In the Crimean campaign it is asserted on eminent French authority that in six months the allied forces lost 50,000 soldiers from disease, and only 2,000 from casualties. In the French campaign in Madagascar in 1894, of the 14,000 men sent to the front, 29 were killed in action, and 7,000 succumbed to disease, most of which was preventable. In our Spanish-American war in 1898, in a campaign the actual hostilities of which lasted six weeks, the deaths from casualties were 293, while those from disease were 3,681, or nearly 14 to 1.

. . . . . . . . .

Perhaps some people might have thought, up to the time of the Russo-Japanese war, that such mortality from disease was unpreventable. But the Japanese have shown us that this view of the matter is not the correct one. Medical men knew this before, but if the public had become quick to become awake to the facts, their representatives in Congress would not have been permitted to go to this late day without enacting laws that would give the required relief. From February, 1904, to May, 1905, there were 52,946 deaths in the Japanese army from casualties and wounds, while the total deaths from sickness amounted to 11,922. This record is unparalleled and unapproached in the history of warfare, and

---

*Editoral in *Medical Sentinel*, Portland, Ore., December, 1905.

merely shows what can be accomplished by intelligent and adequate medical organization. One writer, in answering the question as to how this was accomplished, says it was done in three pre-eminently fundamentul ways. First, through preparation and organization for war such as was never before made in history, as to the medical department; second, through the simple, non-irritating, easily digested ration furnished the troops; and, third, because of the brilliant part played by the members of the medical profession in the application of practical sanitation and the stamping out of preventable disease in the army. Japan organized the medical department of her army on broad, generous lines, and gave its representatives the rank and power their great responsibilities merited, recognizing that they had to deal with a foe which history has shown has killed 80 per cent. of the soldiers who have died, in other wars. As this author pertinently remarks: "She even had the temerity (strange as it may seem to an American or English army officer) to grade her medical men as high as the officers of the line, who combat the enemy who kills only 20 per cent., and to accord them equal authority, except, of course, in the emergency of battle, when all the authority devolves as it should, on the officers of the line. In her home land she organized the most splendid system of hospitals that has ever been devised for the treatment of the sick and wounded, and with her army at the front, she put into execution the most elaborate and effective system of sanitation that has ever been practiced in war."

. . . . . . . . .

It is a sad pity that the near relatives of the unfortunate soldiers who die of preventable diseases, can not be organized, so that they might with greater force demand that their representatives in Congress amend the present inadequate laws. When deemed necessary, tens of thousands of dollars will be expended, and that properly, by the state, in the prosecution of a criminal who has committed one murder. The theory of the prosecution is that life is the most precious possession of man. And yet where not one victim is involved, but tens of thousands, appropriations and salient measures are withheld, because a few paltry tens of thousands of dollars are involved. Our soldiers, in times of war, are slaughtered ease by the thousands. After the turf is laid upon their last resting place, a headstone is placed to mark the spot, the world moves on as before, nobody is punished, and no united effort is made on the part of mourning relatives to secure a reform. The father sheds a tear over the body of his promising son, a victim of typhoid fever. He has, perhaps, no other son to give to the remorseless juggernaut of disease; or he fondly hopes, if he has other sons, that no call for their services will be made by the country. The mother, who has, perhaps, lost her sole support, has no voice in the affair of the nation, and would not know how to exercise it to advantage if she had such a voice. The newspaper reader who learns of the horrible decimation in the camps that have been selected by army officers, without consulting the medical department, and often against their ineffectual protests, is shocked for a few brief days, and acts as if disease were a visitation of God, for which there was no remedy, and no means of prevention.

. . . . . . . . . .

In the meantime, the Medical Department of the Army, staggered and disheartened by the inadequacy of the law, are still striving, year after year, to remedy the present unfortunate state of things. They realize that the conditions existing in the United States, while no worse, perhaps, than in some other countries, are, as regards effective organization, deplorably behind Japan, and disgracefully behind what they should be in such an enlightened country as this. The medical profession of the country can do much to secure an improvement of the status of the Medical Department of the Army. Individual members of the profession can reach their congressmen and senators, whose interest would be valuable. Where they are lukewarm, their interest should be awakened. When war comes, this is a matter of more than general interest; it touches every locality.

. . . . . . . . . .

The army to-day is officered for a strength of 100,000 men ex-except the Medical Department, which is only sufficient for 42,-000. As the Surgeon-General has succinctly put the case: "The three primary duties of the Medical Department are (1) to preserve the effective strength of armies by military sanitation; (2)

to care for the sick and wounded; (3) to conduct the administrative work of the department. To carry out these objects requires a highly specialized and complex organization, and a numerous trained personnel. Military sanitation is now recognized to be a well-marked specialty in medicine, of which the average practitioner knows little more than he does of the methods of military medical administration. The second duty is that for which civilian physicians can be used to advantage, while the first and third must be, in the main, in the hands of trained medical officers in order to secure efficiency, and to relieve the volunteer medical officer of the 'red tape' which, while a necessary evil, is so burdensome and incomprehensible to men not trained in Government methods."

. . . . . . . . . . .

The bill which is now before Congress provides for an increase of medical officers from 320 to 450, exclusive of the Surgeon-General. It reduces the length of service required in the grade of lieutenant before promotion to captain, from five to three years, in order to correspond with the requirements of the naval service. It increases the proportions in the higher grades to that existing for many years prior to the reorganization of 1901, and nearly equalizes them with those existing in the Medical Corps of the Navy. It provides examinations to determine fitness for promotion up to include the grade of colonel. It provides a Medical Reserve Corps which shall constitute an eligible list of medical officers who shall have been examined, and commissioned, if found fit, for service as medical officers with the rank of first lieutenant in case of war or other emergency. These reserve medical officers are intended to replace the contract surgeons, upon which the Medical Department depends for expansion when its numbers are found to be inadequate for the needs of the service, as, for example, when the army is raised to its maximum authorized strength of 100,000 men. They are not intended to in any way replace the volunteer medical officers.

. . . . . . . . . . .

President Roosevelt is fully alive to the importance of this matter, and in a message to Congress last January, he said: "Not only does a competent medical service, by safeguarding the health of the army, contribute greatly to its power, but it gives to the families of the nation a guaranty that their fathers, brothers and sons who are wounded in battle or sicken in camp shall not only have skilled medical aid, but also that prompt and well-ordered attention to all their wants which can come only by an adequate and trained personnel. I am satisfied that the Medical Corps is much too small for the needs of the present army, and therefore very much too small for its successful expansion in time of war to the needs of an enlarged army, and in addition to furnish the volunteer service a certain number of officers trained in medical administration. . . . If the Medical department is left as it is, no amount of wisdom or efficiency in its administration would prevent a complete breakdown in the event of a serious war."

---

### THE MAINTENANCE OF ASEPSIS.

BY DANIEL H. CRAIG, M. D., BOSTON.

There are many surprising happenings in the practice of asepsis as distinguished from the theory. While it is difficult indeed in these days to find the practitioner who is not well versed as to the value of asepsis in the abstract, it is even more difficult to find one who is at all competent to adequately practice such asepsis unless he has served an apprenticeship of greater or less duration under some practical teacher. In other words, I feel that I am justified by experience in making the statement that an ability to maintain a rigid asepsis can not be acquired from reading alone.

Before going further, permit me to explain that I do not believe that absolute asepsis of living tissues is or can be obtained except on possibly rare, accidental occasions. By asepsis, then, as used in the following, I mean the reduction of pathogenic organisms to so small a number that they shall not constitute a morbific, infectious dose for the individual patient. This dose unquestionably varies not only in different individuals, but also widely in the same individual under varying conditions, and therefore the only positive insurance we possess against a morbific infection is the invariable establishment and *maintenance* of the most absolute asepsis possible.

It is not my intention to exploit any particular method of sterilization either for the living tissues or for the inanimate accessories of the operation in this place beyond asking those practicing such branches of medicine and surgery as demand asepsis to adopt the simplest, easiest methods which have been shown to be both bacteriologically and practically efficient. It is here, as nowhere else in life, that cleanliness is godliness and no elaborate processes are either necessary or desirable. It is my belief that antiseptics, with the possible exception of sixty per cent. alcohol, should be absolutely foresworn in the disinfection of living tissues and in the operating room. The long established and too long cherished use of the corrosive sublimate solution in the operating-room as a hand wash is a delusion. No one will deny to corrosive sublimate its well-deserved position at the head of the list of chemical disinfectants, or germicides, but everybody must concede from the reports of all bacteriological experts that it takes time for corrosive sublimate solutions to penetrate the capsule and destroy pathogenic organisms. Granting this, corrosive sublimate solution is in no sense superior for rinsing away infectious material adhering to any surface, hastily, during the course of an operation, to plain sterile water. On the contrary, since by its employment the wet hand may convey an irritating chemical solution to the sensitive peritoneum or other equally sensitive surface upon which it may engender irritation, it is emphatically inferior to the plain sterile water. If we could allow the corrosive sublimate solution the time requisite for full action and then rinse away any remaining impurity with the sterile water, it would be of the greatest possible value. But even the minimum time admitted by bacteriologists as requisite would, in the midst of an operation, be absurd.

Since, therefore, we may not, except at the expenditure of a prohibitory amount of valuable time, restore a lost asepsis, the importance of the maintenance of the original asepsis is manifest, and it is to stimulate to this achievement that I am making this effort. Perhaps nothing will better serve to this end than the citation of a few illustrative examples of the ways in which I have personally observed the defeating of asepsis.

About a year ago it was my privilege to visit one of the largest hospitals of a neighboring city and witness the operating. During the course of a difficult appendicitis operation the surgeon decided to dispense with his rubber gloves, with the use of which he was not fully familiar. Stripping off the gloves, he, with his asepticized hand (and it had been excellently prepared), turned on the faucet, rinsed his hands, turned off the faucet, rinsed his hands for a moment only in corrosive sublimate solution, and proceeded with the operation. All this occurred with nurses and assistants in abundance in the operating-room, any one of which could have saved him the necessity of the slightest contact with any unsterilized object. The dose of pathogenic material obtained from this contact with nonsterile material may or may not have been sufficient to constitute a morbific infection for that particular patient, but it certainly destroyed in one instant the results of many minutes of painstaking preparation. The patients "resistance" is an unknown quantity which for safety's sake must always be considered as at a minimum.

Another illustrative instance occurred in a small rural hospital. The nurse, after elaborate hand sterilization, was not provided with a sterile gown, the operator and his assistants being thus provided, and when her hands were not in use she folded them behind her back, allowing them to rest upon her uniform, which was worn in all her work and must have been far from sterile. In this same operating-room the field of operation was surrounded only by four small towels, no sterile sheets being used to cover the upper and lower portions of the patient, with the result that the patient, coming partly out of the anesthetic, put her own hand into the wound and against the intestines. With regard for the maintenance of asepsis equal to the effort originally made to secure the sterility of all the parts this accident could not have happened.

A third instance, and the last I shall cite, occurred recently in my own operating room. After the patient was ready for the operation to begin a sterile sheet was thrown over the entire upper part of her body, being carried up over her face far enough to screen the abdominal wound from saliva or other contamination if, under ether, she should cough or vomit. The patient's physician, standing beside the table, and whom I know to be a progressive and careful man and one who certainly understands the theoretical side of asepsis thoroughly and whose alma mater and date of graduation testify to the soundness of his teaching on the subject, carefully folded that sheet back so as to bring the parts which he had handled with his unsterilized hands into close proximity with the wound, necessitating, in the interests of the maintenance of asepsis, the substitution of a new sheet.

No one of these occurrences may have done any serious harm to the patient in question, but they were full of possibilities and certainly if left uncorrected would have placed an additional tax upon the patient's powers of "resistance." And speaking of "resistance" leads me to reiterate the belief that, since the establishment and maintenance of absolute asepsis are impossible to living tissues, the so-called "reaction" temperature seen on the postoperative charts of surgical patients is in the vast majority of all cases, especially those in which no demonstrable degree of surgical shock obtains, neither more nor less than an index of the amount, or dose, of infectious material introduced.

It is, then, my desire now that practically the entire profession realize and concede the value of asepsis and have learned how, by one means or another, to acquire a practical degree of sterility, that they now turn their attention to learning to maintain that sterility to the very end of its need; for believe me, except in the trained operator, and not always there, as shown in the foregoing instances, this ability is rarely seen. It is fortunately an acquirement which does not lengthen but rather shortens every operation and requires only presence of mind sufficient to cause one to draw back his sterile hand, gown, or instrument from contact with an unsterilized object as he would his hand from a red hot coal. It means the acquirement of the "aseptic conscience" and its lack should be supplied along with the knowledge it enhances, for its acquirement is as easy by practice as in any other clinical or surgical manipulation. —*New York and Phil. Medical Journal*, Jan. 20, 1906.

---

Tinnitus aurium, present only in the recumbent posture, is suggestive of aneurism of one of the posterior cerebral vessels.—*American Journal of Surgery.*

### APPENDICITIS IN CHILDREN.

Dr. J. H. Hess (*Archives of Pediatrics*), suggests the following points of diagnosis :

1. Spontaneous pain manifested in the very young by fitful crying and sleep. In typical cases it is at first localized at "McBurney's point," but it soon becomes diffuse, radiating from the umbilicus into the pelvis. Young children complain of "bellyache" and usually lie curled up on one side. More rarely the pain may be referred to the right testicle or to the neck of the bladder, because of the close relation with the right ureter, a point which should be carefully borne in mind while operating. In rare instances we may have perforation before we get a history of pain. (Spieler.)

2. Hyperesthesia—tenderness on pressure is apt to be difficult to localize in the young, and therefore of little value.

3. Muscle spasm or rigidity of the right rectus is present unusually early in children, but it is difficult to elicit and localize, because of the child's fear of examination and its tendency toward rigidity of the abdominal muscles in general. Its elicitation is one of our most valuable diagnostic aids and is a fitting reward after a gentle and patient examination.

These three symptoms when associated with vomiting and constipation, make a positive diagnosis possible in at least a majority of cases of older children, but in infancy and early childhood the diagnosis becomes far more difficult because of the tendencies of the child to evade examination, and it becomes necessary to look for further aid.

4. Nausea and vomiting are usually present a short time after the onset of pain; they usually cease as soon as the stomach is empty, but reappear in the later stages of the disease when perforation has occurred and abscess formed or an intestinal paresis exists.

5. Chill—rarely seen in the first stage.

6. Pulse—in simple cases the pulse usually corresponds with considerable regularity to the temperature, even more so in children than in adults; also as long as the process remains localized; when extensive peritonitis exists the pulse becomes rapid and weak easily comprehensible and irregular in character.

7. Temperature is unreliable, as the worst types may run their course without any great rise of temperature after perforation and beginning peritonitis. Rectal temperatures only are reliable.

8. Constipation is the rule in these cases. Diarrhea may be present, and the latter cases as a group are less urgent than those associated with constipation, whose early occurrence usually indicates intestinal paresis. The absence of blood, bloody mucus, etc., is important, because of their predominance in intussusception.

9. Tympanites, with gradually-increasing persistent abdominal distention is usually a later development.

10. Flexion of the thigh, which is usually well brought out by attempts at extension and flexion of the two thighs.

11. Unconscious tendency to place the hands in the region of the appendix to prevent examination of the sensitive area, which, unfortunately, is not always located in the classical region, is of frequent enough occurrence to be of diagnostic value.

12. Rectal palpation—in older children whose attention can be claimed, this method of examination is of prime importance. In catarrhal cases I have several times been able to locate the point of the greatest pain with accuracy, and not infrequently the outline of the inflamed appendix can be palpated through the thin rectal wall in these small pelves. In later stages its value for locating localized abscesses or intraperitoneal pressure is of inestimable value.

13. Tumor by abdominal palpation, if the illness is of several days' duration, associated with a septic temperature, rigor, luekocytosis, etc., should always lead to the consideration of appendicitis.

14. Increased frequency of micturition, due probably to the close proximity of the right ureter and bladder to the area of inflammation.

15. Leukocyte count—as a rule, in children it is not as valuable as in adults, because of the great range under normal conditions, going to 20,000, or even higher. Probably the most reliable finding is a decided leukopenia of 5,000 to 8,000 in the presence of peritoneal involvement, which indicates either a low resistance or a preponderance of the infection over the natural resistance, and is always an evil omen. Probably more important than a simple increase will be a differential count when better understood. Gudohin estimates that in infancy polymorphonuclear cells average 34.0 per cent., monoclear lymphocytes 59 per cent., and transitional forms 6.4 per cent., while in later childhood the polymorphonuclears increase more nearly to the normal. Remembering these characteristics a leukocyte count of 30,000 or more, with 80 per cent. polymorphonuclear cells, or 15,000 with 90 per cent. polymorphonuclear cells, should always lead to the suspicion of the presence of pus. In intestinal obstruction the leukocytes number even 50,000 to 80,000, which must not be forgotten. In all cases the accompanying findings must be considered.

16. Iodophilia test, which, in the presence of pus, has some claim to recognition. My own experience with it has not been entirely satisfactory. Although the laboratory findings are of considerable value to the diagnosis of atypical cases, and aid in the prognosis of the most virulent types, they should not, except in rare instances, be allowed to set the time of operation. Therefore, to be of value, we should (1) count the number of leucocytes; (2) make a differential count, remembering the difference between the normal infants and adult count; (3) stain blood film by special methods. Iodin reactions, etc.

17. History of previous indefinite attacks of abdominal pain associated with a tendency to persist, and a slow retrogression of the group of indefinite symptoms, is of vast importance, and the history should always secure careful consideration.

---

### ON CONVULSIONS IN EARLY INFANCY.

In speaking of the etiology of convulsions occurring in early infancy, John Thompson (*Practitioner*, October, 1905), divides it into the predisposing and exciting causes as follows: Of predisposing causes four may be mentioned: (1) There is, of course, the age of the patient—the state of development of the infant's nervous system, predisposing him to all kinds of convulsive attacks. (2) Certain general diseases may predispose—especially rickets. This is by far the most important of the predisposing causes, as it is the only one which is amenable to immediate treatment. The tendency to convulsions, in rickety children, rapidly disappears under antirachitic treatment, even although obvious sources of peripheral irritation persist. (3) A very important predisposing element is inherited nervousness of constitution. Some children are hereditarily so nervous that any rise of temperature or any peripheral irritation, however slight, may bring on a fit. This state of nervousness may be found in children who seem otherwise strong; and sometimes many members of the family may have it to a marked extent. (4) Another predisposing cause is a permanently damaged state of the brain from any cause—quite apart from any recent changes in it. An area of cortical sclerosis, for example, even when it does not affect the mental functions, is very often accompanied by a tendency to convulsions. In the same way, nearly all the development and other lesions, which produce imbecility, predispose also to convulsions on slight provocation. The recurrence of convulsions in very young children should, therefore, always lead to a careful investigation of the child's mental state. Possible exciting causes are very numerous indeed. The most important of them may be classed in one or other of three groups: (1) They may result from a number of intracranial causes (diseases, injuries or circulatory changes). Such, for example, are, concussion, hemorrhage, tumor, abscess, and meningitis of all kinds; also cerebral congestion, which we sometimes get in whooping cough, and in some cases of congenital heart disease, and the cerebral anemia, which accompanies severe diarrhea and loss of blood. (2) General acute morbid conditions, again, are often responsible for a convulsive attack. A sudden rise of temperature, such as would produce a rigor in an adult, will often in an infant cause a convulsion. This is seen in pneumonia and in various of the exanthemata, especially in scarlet fever. In uremia, fits are not uncommon, and a large number of poisons, both metallic and vegetable, often occasion them. (3) Peripheral nervous irritation is certainly a common exciting cause of fits, and one which is often present even when its site of origin is obscure. Undigested matters in the bowel or stomach, painful lesions connected with dentition, otitis, or phimosis, may readily start a convulsion in rickety or neurotic infants. Generally, as mentioned above, the place of rigors is taken in young children by convulsions. It is, however, a very curious thing that acute irritation of the renal pelvis frequently gives rise to rigors, even in young babies. Among twelve cases of acute pyelitis from *Bacillus coli* in infants, that I have seen, no fewer than six suffered from rigors. Two of the cases were in children of five and seven months, respectively. In none of them have there been any ordinary convulsions.

---

### TWENTY CASES ILLUSTRATING THE FREQUENCY OF MISTAKES IN THE DIAGNOSIS OF PREGNANCY.

From an interesting review of these case-histories, T. M. Burns (*The Colorado Medical Journal*, July, 1905,) concludes that they demonstrate the frequency of mistakes and illustrates:

1. Unpardonable error.

2. The necessity of an examination when abnormal symptoms are present, and the fact that subinvolution and pregnancy may co-exist.

3. The value of special symptoms.

4. The fetal movements may disappear, the abdomen diminish in size, and pregnancy nevertheless continue to a normal labor.

5. That, to the experienced examiner, a two-months' gestation may produce no apparent increase in the size of the uterus or any other definite sign of pregnancy.

6. That a multipara may be pregnant and in labor and not know it.

7. That in tubal pregnancy the fetus may die early, the placenta grow to term and cause very few symptoms.

8. That in salpingitis a large pus cavity may exist in one broad ligament and an extra-uterine pregnancy in the other.

9. That adhesions of the omentum, the result of an extra-uterine pregnancy, may simulate a modular fibroid uterus.

10. That a thick-walled seven-months' pregnant uterus may be mistaken for a dermoid cyst of the ovary.

11. That a five-months' pregnant uterus may resemble a tumor even after the abdomen is opened.

12. That a woman may be in labor and be treated for something else with fatal result if an examination is not made.

13. That even after the fourth month some physicians say that there are no signs of pregnancy, either through lack of skill or to prevent criminal abortion.

14. That a distended bladder during pregnancy may raise the uterus and itself high in the abdomen and produce signs of twins, a double uterus, an extra-uterine pregnancy, or pregnancy complicated by a cystic tumor.

15. That fecal accumulations may distend the bowels so as to produce a tumor that will cause all the subjective signs and some of the objective signs of a five or six-months' pregnancy?

16. That a pregnant fibroid uterus may simulate twins and apparently contain a fetus after one is born.

17. That amenorrhea with an occasional slight discharge of blood in a fleshy woman may be treated for threatened miscarriage if a careful examination is not made.

18. That extra-uterine pregnancy may be diagnosed in normal multiple pregnancy.

19. That ascites may be diagnosed during pregnancy and paracentesis cause death of the fetus.

20. That tuberculosis and a uterus enlarged by a fibroid may closely simulate pregnancy.

---

### FIRST AID TO THE WOUNDED IN NAVAL BATTLES.

Under this title Don Juan Redondo (*Journal of Military Surgeons*) discusses in detail this subject and concudes with the following summary:

1. First aid to the wounded of naval battles is one of the most mportant problems in modern naval surgery.

2. To comprehend the extent of its application it is necessary to fix with exactness the character of the wounds, the peculiar conditions of the modern ship and the elements that we have at our disposal.

3. The wounds occasioned in naval battles are characterized by their great extent, irregularity, multiplicity and the frequency with which fragments of the projectiles and foreign bodies remain therein. Practically they are septic wounds. Burns, varied traumatisms and shock often complicate them.

4. The interior arrangement of the modern man of war creates great difficulty in the application of quick and efficient aid to the wounded during battle.

5. The sanitary service during the fight must be in keeping with the actual arrangement of the modern war ship. The greatest obstacle to the solution of the problem that we are studying lies therein.

6. A good sanitary organization must be careful of the wounded since the man is put *hors de combat.*

7. The wounded must receive some aid on the spot where he falls.

8. Every combatant must be provided with one appropriate "individual dressing." Though its value is very limited, it should not be dispensed with.

9. The "relief stations" are the point of connection between the deck and the dressing station. Their utility is less than is generally believed. It is sufficient, however, for a man to be saved in them, to entirely justify their existence.

10. The wounded can only receive efficient aid at the hands of the surgeon.

11. The dressing station is the fighting post of the surgeon. In it is where he can and ought to care successfully for the wounded.

12. As a general rule it must be established that the surgeon should limit himself during action to fulfilling purely vital indications; it must be his principal mission to stop hemorrhage, cover the wounds with a simple dressing and revive those who are victims of syncope or shock. This rule must not be so absolute as not to permit the practice of important operations when circumstances will permit.

---

DO NOT empty a thyro-glossal cyst by aspiration before extirpating it. It is well to inject the cavity with a methylene blue solution first, in order to make sure that all parts of the cyst wall will be extirpated. Another method is to first empty the cyst and then fill it with paraffin.—*American Journal of Surgery.*

### THE RELATION OF THE KIDNEYS TO ECLAMPSIA.

In discussing the relation of the kidneys to eclampsia P. K. Brown, *Jour. A. M. A.*, January 13, 1906, gives the following summary.

1. Albumin is present in fully eighty per cent. of normal pregnancies.

2. Albumin and casts are found in at least thirty per cent. of all pregnancies.

3. There is no reason to suppose that the renal condition thus revealed is the cause of eclampsia.

4. That there is some connection, however, between the albuminuria and the extrarenal cause of the eclampsia is likely, in view of the nearly constant association of the two.

5. It has been shown that neither any normal end product nor any known intermediary product of metabolism is the cause of eclampsia.

6. It is reasonable to suppose that deficient thyroid or parathyroid actively plays a part, at least, in some of the cases of eclampsia.

7. There are probably several casual factors to the separate or concomitant action of which eclampsia seems to be due.

8. The most significant experimental work done up to this time points to the fact that in the placenta are formed the toxic substances which probably are responsible for eclampsia.

9. There is good evidence that these same substances are, in all probability, the causes of the headache, edema, abdominal pain, and particularly the albuminuria present in non-eclamptic and non-nephritic cases.

10. The nervous systems of pregnant women are in an abnormally sensitive condition. Their blood has been shown to be abnormally toxic. There is no conclusive proof of an increased or diminished toxicity of the urine.

11. My own experiments have shown that the muscles of pregnant guinea-pigs can be thrown into convulsions more easily than those of non-pregnant animals.

### ADMISSIBILITY AS EVIDENCE IN CRIMINAL PROCEEDINGS OF BLOODHOUNDS FOLLOWING TRAIL (NEBRASKA SUPREME COURT).

George W. Bratt was convicted of burglary. The Supreme Court reversed the judgment, as stated in No. 97 of the N. W. Reporter, page 593. The crucial point in this case was the admissibility of the evidence of the conduct of bloodhounds in following the trail of the burglar from the scene of the crime to the defendant's residence. The court holds that the evidence was improperly submitted. The court stated that there is a prevalent belief that in the pursuit and discovery of fugitive criminals the bloodhound is practically infallible; that it is a commonly accepted belief that he will start from a place where a crime has been committed, follow the track for miles after he has been given the scent, find the culprit, confront him and, mirabile dictu, by accusing "bay and mien" declare "Thou art the man!" The strange misbelief is in the minds of some people apparently a deep-rooted fact, and it is a delusion which abundant actual experience has failed to dissipate. It lives on from generation to generation. It has still the attractiveness of a fresh creation. "Time writes no wrinkles on its brow." The court stated that the path of every individual from the cradle to the grave is strewn with putrescent excretions thrown off from the body and this matter is in a process of rapid decomposition. The bloodhound, which has great ability for distinguishing smells, follows the odor thus generated, and for a short time a man may be easily trailed in the woods or open country. But in the city, after the lapse of considerable time, as in this case—about twelve hours of sunlight, where the trail is crossed by hundreds of others, the work is obviously very difficult, yet the dog does the best he can. Nice and delicate questions are time and again presented to him for decision, and as to considerations which move his choice of paths he can not be cross-examined and the jury informed. The result of all this is that the conclusions of the dog are too unreliable to be accepted by the jury as evidence.—*Medical Fortnightly.*

### X-RAY DIAGNOSIS OF RENAL CALCULUS.

Morton Smart (*British Med. Jour.* Sept., 16, 1905), describes the "Cathode" and X-Rays and discusses the possible errors in diagnosis, which may occur on account of the character of the stone, the size of the patient and the condition of the kidneys. He deduces the following conclusions:

1. That the method is an extremely useful auxiliary to the ordinary methods of diagnosis.

2. That the method should be resorted to in every case of suspected stone, and that no operation should be decided upon until the case has been carefully photographed.

3. That the method entails a great responsibility and should in every case be carried out with the greatest possible care, for the patient's sake as well as for the operator's reputation, as a mistake may lead to such serious consequences.

4. That the great increase in the power of the apparatus used, and the increased knowledge of how to obtain the best results, will soon enable X-ray specialists to exclude all doubt in the interpretation of a negative.

5. That in cases where the negative shows the shadow of a stone and an operation is decided upon, the patient should be rephotographed under as nearly as possible similar conditions a day or so prior to the operation. I consider this necessary, because in one case a patient was photographed and a diagnosis of renal calculus was made. The operation took place some time afterwards, and no stone was found in the kidney. Another photograph was taken shortly after the operation, and the stone was again seen, but this time it was in the ureter. During the interval between taking the photograph and performing the operation the stone had moved downwards.

6. Lastly, I think every case should be taken stereoscopically at least once.

---

ATTACKS of abdominal pain preceded by "rumbling" of the bowels is suggestive of some obstructive condition.—*American Journal of Surgery.*

### THE SURGICAL TREATMENT OF TUBERCULOUS LYMPH NODES.

Chas. N. Dowd (*Annals of Surg.*, July, 1905) points out that: Tuberculosis of the cervical lymph nodes is apparently due to infection received from the fauces, pharynx, or nasal mucous membrane, in the majority of cases. The disease shows a tendency to extend to the lungs and other internal organs. Statistics indicate that such extension occurs in one-quarter to one-half of the cases in which the nodes are not removed. Entirely apart from its tendency to infect other organs, the disease is very tedious, causes great discomfort and disability, and leaves disfiguring scars. The thorough removal of the disease nodes by operation has given better results than any other method of treatment which the writer finds recorded. The records of operation justify the following assurances: (a) In favorable cases: Safety of operation (many operators reporting more than one hundred cases without mortality); a scar which is hardly to be seen; probable confinement to bed for two or three days; the wearing of a bandage or dressing from one and a half to three weeks; freedom from recurrence in about seventy-five per cent., and ultimate recovery in about ninety per cent. of the cases. (b) In the less favorable cases: Safety of operation; less disfigurement from scars than discharging sinuses will cause; freedom from recurrence in fifty to fifty-five per cent., and ultimate cure in seventy to seventy-five per cent. of the cases. Transverse incisions, either in the neck creases or parallel to them, are usually to be used. They should be so placed that the fibers of the facial nerve will not be cut. A vertical incision back of the hair line is occasionally helpful. Extensive incisions are necessary for the far advanced cases. It is not feasible to divide the cases into groups, some suitable, others unsuitable for operation. Every case with tuberculous cervical lymph nodes should be operated on unless there is a particular reason to believe that the operation would not be endured.

---

SEVERE and repeated headaches may be due to the unsuspected presence of otitis media, with or without mastoiditis.—*American Journal of Surgery.*

### THE ETIOLOGY OF ECLAMPSIA.

After discussing the various theories of eclampsia Hugo Ehrenfast (*St. Louis Courier of Med.*, Sept., 1906) says:

"In concluding my remarks I wish to point out briefly a few of the mistakes that are commonly made in the consideration of the causation of eclampsia. There seems to exist a great deal of confusion between cause and effect. Many of the pathologic changes described in kidneys and liver are not so much the cause as the result of toxemia, and only secondarily, by way of the vicious circle which I have endeavored to explain to you, help to exaggerate the existing toxemia. Another mistake which is very prevalent, is, that altogether too much stress is laid upon the importance of the kidneys. I have demonstrated that the kidneys certainly are not the only organs which by elimination prevent a pathologic accumulation of toxins. The function of liver or thyroidea are just as important in this respect. Of course, the urine offers an easy means of ascertaining the conditions of the kidneys, and therefore, it is so much easier to recognize an alteration in their function. But I think it is absolutely useless to fight about the question which particular renal lesion is chiefly responsible for eclampsia. All is dependent upon the total work of all the eliminating organs, and if in some cases of eclampsia, no renal lesion can be detected, and no albumen is found in the urine, then probably in these cases the eliminative action of liver or thyroidea or another important organ is absolutely inefficient, or possibly, by some process still unknown to us, a very large amount of toxic material was suddenly thrown into the maternal circulation, too large to be taken care of by the 'organs of defense.'"

---

WHEN performing amputation, arthrectomy, osteotomy or similar operation it is wiser to leave the constrictor in place until the dressing is partly, or entirely, applied, than to remove it after tying the large vessels, in an effort to secure the small ones. In the former case the snugly applied dressing will safely prevent hemorrhage; in the latter case, there may be an alarming loss of blood from the numerous small vessels in the very time the efforts are made to tie them all.—*American Journal of Surgery.*

### THE PHYSICIAN AS A BUSINESS MAN.

Rightly or wrongly, the physician is regarded as by no means acute in business matters. That he is looked upon as not "a man of affairs" is evidenced by the manner in which the "get-rich-quick" fraternity— or "con"-fraternity—pester him. He is an object of their most unremitting attention, which fact, in itself, is a more or less conclusive proof that the medical man is somewhat childlike as regards the art of money-making outside of his own profession.

The members of the medical profession, as a rule, plead guilty to this indictment, although it must be borne in mind that there are some very notable exceptions. There are medical men in this country who are as keen and capable money-makers as members of any other class. On the whole, however, it must be admitted that the doctor parts with his hard-earned coin to the wily promotor with surprising credulity. In the New York *Medical Journal* of a recent date, Dr. Frank Lydston discusses this matter editorially. His article deserves to be read carefully and inwardly digested by every physician in the United States. He describes vividly many ways in which the unworldly doctor is induced to enter into bogus schemes and gives the following excellent advice to his brother practitioners. He puts it in the form of certain fundamental facts of finance:

1. The man who has a profitable mining, land, or agricultural scheme to finance is not compelled to go into the office buildings of our cities, or to the modest dwellings of our country practitioners, for the purpose of inducing them to invest their hard-earned dollars. Had I a really excellent mining or other scheme to promote, I think I could spend my time much more profitably among capitalists than among doctors or other professional men. The amount of unused capital in America that is lying in wait for judicious and profitable investment is so great that even the fools should be able to understand the situation.

2. The legal rate of interest is established by our general financial and commercial conditions. This legal rate is supposed to be a safe rate. Yet our men of great wealth, when they desire to make investments, buy Government bonds, the interest upon which is about half the legal rate of interest. They do this because they consider the bonds the only investment which is absolutely safe. This is a hint that the physician who has a few hard-earned dollars which he is tempted to invest in wildcat schemes would do well to remember.

3. The most important point is this: With every 1 per cent. of interest promised above the legal rate, the danger compounds. The difference in safety between an investment which promises 5 or 6 per cent. and one which, it is alleged, promises 10 per cent., is something staggering.

4. It is well to remember that most of the men who have lost money in speculation have done it while "backing another man's game." The professional gambler considers this a very dangerous practice. As he expresses it, "Something always goes to the 'kitty,' whose remorseless maw will inevitably break the outsider if he plays the game long enough." The little commission charged for turning deals on the stock exchange or grain market is the equivalent of the amount that is paid to a "kitty" in a gambling game. This amount, trivial as it is, constitutes terrific odds against the man who is playing the game from the outside. It is the fleece of which the lamb must inevitably be shorn in time.

Why the physician should be generally so incompetent in business affairs out of his own field of work is a question hard to answer offhand. His profession, perhaps, is so exacting that it leaves him little time to attend to other matters. The work of a doctor, too, brings him into contact with so much suffering, that it is apt to make him unworldly and trusting. Again, it is more easy to gain an audience with a physician than with members of other professions, or business men. Be the reason, however, for the gullibility of the physician what it may, the fact remains that he is particularly open to the art of the fakir and swindler. There are signs that at last his eyes are being opened, and that in the future the doctor will lend a deaf ear to the wiles and blandishments of the mining promoter and others of that ilk.

### RULES OF HEALTH.

A famous New York physician, now hale and handsome at seventy-five, sums up his half century of medical practice and observation in these simple rules of health:

1. Be temperate in all things, in matters of amusement or study as well as in regard to foods and drinks. To be temperate in all things, however, does not imply that one must be a prohibitionist about anything.

2. Don't be afraid to go to sleep, for sleep is the best restorer of wasted energies. Sleep a certain number of hours every night, and then remember that a short nap during the day is a safer rejuvenator than a cocktail.

3. Don't worry—either about the past or the future. To waste a single hour in regret for the past is as senseless as to send good money after that which has been irrevocably lost. To fret one's self about what the future may have in store is about as reasonable as to attempt to brush back the tide of the ocean with a broom. Worry of whatever kind, banishes contentment, and contentment is a necessity of youth.

4. Keep the mind youthful. Live in the present with all the other young people. Don't get to be reminiscent. Let the old people talk about the past, for the mere act of thinking about old things reminds the mind of its years. Reminiscences are dangerous—whether they be soothing or sweet or sad—for they characterize old age, and must be sedulously avoided by those who would be ever young.

5. Keep up with the times. To accomplish this learn one new fact every day. The mind that is satisfied to live upon the lessons it learned in its youth soon grows old and musty. To keep young it must be fresh and active—that is, abreast with the times. The old methods of thought and the old facts may have been correct enough once upon a time, but that time has passed. To-day they are obsolete and only amusing as relics of antiquity. To remain young, therefore, one must keep the storehouse of the memory clear of all such rubbish. Throw away one of the mildewed relics every day and replace it with some newer, fresher and more up-to-date fact.

Here, then, is this New York physician's secret of perennial youth in a nutshell:

Be temperate! Don't be afraid to go to sleep! Don't worry! Keep the mind youthful! And—keep up with the times!

It is not a difficult rule of life to follow. It is ever so much easier than wandering about strange lands in search of hidden springs. It is somewhat pleasanter than stewing over ill-smelling crucibles. Moreover, it has the advantage of being thoroughly practicable, which makes it worth trying.—*Montreal Pharmaceutical Journal.*

---

### SUCCESSFUL TREATMENT OF LEG ULCERS.

To ascertain the cause in the treatment of leg ulcers is of greatest importance. A tuberculous, diabetic or syphilitic ulcer will require much closer study as to the constitutional condition of the local treatment. Anything interfering with the venous flow, such as constipation, must be immediately corrected, and the patient's general nutrition looked out for. The leg should be rendered surgically clean by the generous application of sinol soap, followed by irrigation of Thiersch solution. No matter what the cause of the ulcer be, it is wise, where possible, to confine the patient to bed with the foot elevated during the course of treatment; the limb should be firmly bandaged, extending from the toes to a point several inches above the ulcer. If possible, excision of the veins of varicose ulcer should be performed. Ulcers covered with unhealthy granulating surface or sloughing edges should be curetted, after which thoroughly irrigated with Thiersch solution and dressed every twenty-four or forty-eight hours with a hot Thiersch pack? When the surface presents granulations, applications of bovinine pure should be made, changing them three times in twenty-four hours. The most careful toilet of the limb should be made at each dressing. As a rule, the basis of all chronic ulcers is made up of an unhealthy, granulating mass, consequently it is impossible to bring about a cure until this has been removed. It will be readily appreciated that an ulcer thus covered can not absorb, consequently the great nutritive properties contained in bovinine can not be effective. This mode of treatment may be applied successfully to any form of ulcer, no matter what its cause may be.—*Ryle, The Medical Fortnightly.*

---

### NEISSER'S RESEARCH ON MONKEYS IN THE EAST INDIES.

The *Deutseh med. Wochft.* is publishing Neisser's report of his months of research in the Dutch East Indies. He had the largest number of monkeys at his disposal that have ever been collected for a single purpose, a total of about 900, including eleven orang-outangs, from twenty to thirty gibbons and the rest smaller monkeys. He was particularly impressed with the morbidity of the monkeys, intestinal diseases, dysentery and helminths making constant ravages among them. He found that there is not a great difference between the smaller monkeys and the orang-outangs and gibbons in respect to infection with syphilis, as has been previously assumed. The higher species are more susceptible, but the infection proceeds about the same in each kind of monkey. All his attempts to preserve syphilitic material for use at a later date has proved fruitless. All virulence had vanished by the sixth or seventh hour after removal from the body. Even from the cadaver, inoculations were always negative after an interval of eighteen hours. Excision of the focus a few hours after infection was unable to arrest the generalization of syphilis, and the most varied attempts to prevent the development of the infection after inoculation never proved successful. The generalization of the infection could usually be demonstrated by the fifty-fourth to the hundred and fortieth day after the inoculation. Simultaneous mercurial treatment never succeeded in preventing the development of the primary lesion, and, in one instance, material from a mercury-treated animal proved exceptionally virulent when inoculated into others.—*Journal A. M. A., Jan. 27th.*

### MODIFIED ALEXANDER'S OPERATION.

Sandberg (*Amer. Jour. Obstetrics*, Sept., 1905,) has devised a modified Alexander's operation to be employed in cases of retrodisplacement with prolapse of more than a moderate degree. He describes his operation and claims for it the following advantages:

1. There is only one incision. 2. That this is located where it gives the best access for examination of the pelvic organs and for work in the pelvic cavity. 3. One opens the abdomen for exploration with much less hesitancy when all work can be accomplished through the same incision, and many of the causes of failure of the Alexander operation may be ascertained and avoided by an exploratory opening of the abdomen. 4. The intra-abdominal work is limited as much as possible, stitching of the uterus or the round ligaments intra-abdominally being avoided. 5. It leaves no adhesion or bands between the abdominal wall and the uterus to interfere with the expansion of the bladder, or growth of the uterus in pregnancy, or to cause obstruction of the bowels. 6. The intra-abdominal relations are left in a condition most closely resembling the normal. 7. The part of the round ligaments retained is the thickest and strongest.

---

### TREATMENT OF POST-ANESTHETIC VOMITING.

Dr. Thomas D. Luke (*Practitioner*, September, 1905), divides the treatment of sickness occurring after anesthetics into (a) general and (b) local. In the first place, a quiet and warm atmosphere is essential in minimizing any unpleasant after-effects of chloroform or ether. A nutrient or simple saline enema is often of service administered directly after the patient has been put back to bed. With regard to local treatment, the mouth may be washed out with an antiseptic fluid. One teaspoonful of Hux-Sal in half a pint of tepid water makes a very agreeable and efficacious solution for this purpose. As a preventive of vomiting, a little egg-white solution, or albumen-water, in which two or three minims of the tincture of nux vomica have been dissolved, and flavored with a little fresh lemon-juice, is often successful. In other cases, a little sodium bicarbonate in plain hot water will dissolve the mucus that clings to the mucous membrane of the upper part of the alimentary canal. The wholesale deprivation of fluids is as cruel as it is unnecessary. Drugs are rarely of service, though minim doses of the vinum ipecacuanha or of tincture of iodine in one drachm of water may be tried in obstinate cases of vomiting. Chloretone has not, in the author's experience, proved to be of much service. A mustard plaster over the epigastrium may relieve the condition when all else has failed.

---

### ECLAMPSIA.

Kirkley (*American Jour. of Obstetrics,* Sept., 1905,) draws the following conclusions:

1. The toxines producing eclampsia consist of waste products from the liver, intestines and kidneys augmented by fetal and uterine metabolism. 2. Renal insufficiency, rather than albuminuria is usually an etiological factor. 3. A causative relation exists between the condition of the urine in pregnancy and eclampsia, because toxemia results when the urea excreted diminishes. 4. Prophylactic treatment, encouraging elimination through the emunctories, is usually successful. 5. Venesection in suitable cases is the best prophylactic. 6. The uterus should be emptied when other means of relief have failed. 7. Venesection in suitable cases is the best curative agent, because of its promptness in removing toxines. Veratrum viride as a substitute is purely visionary. 8. Morphine has no place in the treatment of eclampsia, because it hinders elimination by the kidneys and bowels.

---

### MEDICAL SOCIETY OF THE MISSOURI VALLEY.

The next meeting of this society will be held in St. Joseph, on Thursday and Friday, March 22 and 23, under the presidency of Dr. John E. Summers, Jr., of Omaha. The local arrangements are in the hands of Drs. Jacob Geiger, O. B. Campbell and C. R. Woodson, and hospitable St. Joseph extends a hearty welcome to all.

Among those who will contribute to the program are: Dr. N. S. Davis, Jr., L. L. McArthur and Fenton B. Turck, of Chicago; Dr. S. Grover Burnett, Kansas City; Dr. Chas. H. Mayo, Rochester, Minn.; Dr. C. O. Thienhaus, Milwaukee, Wis.; Dr. D. C. Gore, Marshall, Mo.; Dr. Prince E. Sawyer, Sioux City, Ia.

Those wishing to contribute papers should send in their titles at once, as the list will close February 15.

If you are not a member of this progressive society, now is the time to join. Two meetings a year—initiation one dollar, annual dues one dollar, including the *Medical Herald*.

CHAS. WOOD FASSETT, M.D., Secretary.
St. Joseph, Mo.

---

### A STUDY OF THE BONY PELVIS IN 150 CASES.

The conclusions reached by Effa V. Davis (*Journal A. M. A.*, December 2d) are that:

Deformity occurs often enough to make pelvimetry a practical part of the examination of pregnant women.

Generally contracted pelves form by far the most common deformity in American women, though the rachitic pelvis is often present in those who have been artificially or imperfectly breast-fed in infancy.

Inebriety in the parents is the most constant element toward degenerate types in the deformities studied.

The size of the infant can be regulated by diet and exercise if carried out strictly for a proper time during the last three or four months of pregnancy.

---

THE POPE AND THE MIRACLES AT LOURDES.—Dr. Boissaire of Lourdes, France, sent recently, as usual, his annual report to the Pope of the miraculous cures effected at the famous grotto of Lourdes in his charge. He received, in reply, a letter from Dr. Lapponi, the medical attendant of the Pope, stating that in future the latter wished to have arrangements made to establish the identity of the persons thus cured, with depositions of physicians and witnesses who had seen the patients before their cure, placing the Lourdes experiences on a more scientific basis than hitherto.—*A. M. A., Jan. 27, '05.*

